# Apolipoprotein E Genotype e2: Neuroprotection and Its Limits

**DOI:** 10.3389/fnagi.2022.919712

**Published:** 2022-07-14

**Authors:** Hyun Kim, Davangere P. Devanand, Scott Carlson, Terry E. Goldberg

**Affiliations:** ^1^Department of Psychiatry, Columbia University Irving Medical Center, New York, NY, United States; ^2^Department of Geriatric Psychiatry, New York State Psychiatric Institute, New York, NY, United States; ^3^Department of Neurology, Columbia University Irving Medical Center, New York, NY, United States; ^4^Department of Anesthesiology, Columbia University Irving Medical Center, New York, NY, United States

**Keywords:** APOE e2, Alzheimer’s disease, neuropathology, neuroprotection, biomarkers, cognition

## Abstract

In this review, we comprehensively, qualitatively, and critically synthesized several features of APOE-e2, a known APOE protective variant, including its associations with longevity, cognition, and neuroimaging, and neuropathology, all in humans. If e2’s protective effects—and their limits—could be elucidated, it could offer therapeutic windows for Alzheimer’s disease (AD) prevention or amelioration. Literature examining e2 within the years 1994–2021 were considered for this review. Studies on human subjects were selectively reviewed and were excluded if observation of e2 was not specified. Effects of e2 were compared with e3 and e4, separately and as a combined non-e2 group. Our examination of existing literature indicated that the most robust protective role of e2 is in longevity and AD neuropathologies, but e2’s effect on cognition and other AD imaging markers (brain structure, function, and metabolism) were inconsistent, thus inconclusive. Notably, e2 was associated with greater risk of non-AD proteinopathies and a disadvantageous cerebrovascular profile. We identified multiple methodological shortcomings of the literature on brain function and cognition that could have contributed to inconsistent and potentially misleading findings. We make careful interpretations of existing findings and provide directions for research strategies that could effectively examine the independent and unbiased effect of e2 on AD risk.

## Highlights

- APOE e2 is the only replicated variant identified in the genome associated with longevity, though it is unclear if this is solely due to its effects on reducing AD risk or is partially due to other factors.
- APOE e2 is robustly associated with reduced risk of clinically diagnosed AD and reductions in amyloid plaque and tau Braak stage in neuropathological studies. PET amyloid imaging and CSF Ab42 levels are consistent with the pathology findings.
- Remarkably, there were sharp limits to e2 neuroprotection against other proteinopathies; e2 may promote some tauopathies and fronto-temporal lobe dementias (e.g., Pick’s, progressive supranuclear palsy) and some types of cerebrovascular disorders (e.g., lobar hemorrhage due to cerebral amyloid angiopathy).
- Both e2 isoform conformation and protein abundance may account for some of its neuroprotection, with higher ApoE protein levels associating with more favorable outcomes.
- In structural MRI studies, e2 was associated with greater hippocampal volume in late life. In contrast, e2 was associated with greater white matter hyperintensity burden (a measure of microvascular disease).
- Cognitive findings were inconsistent, though they generally favored e2 when present and when studies were longitudinal. Nevertheless, it cannot be said that e2 is a general cognitive enhancing gene variant across the lifespan. Findings from functional MRI studies were quite disparate. It remains an open question whether e2 impacts specific neural circuitry.

## Introduction

The gene APOE is triallelic with variants arising from amino acid substitutions in the gene’s exon 4: e2, e3, and 4. APOE e4 has been the focus of apolipoprotein E genotype (APOE) investigation since Allen Roses discovered its association with Alzheimer’s disease (AD) in 1993 (Corder et al., 1993; Strittmatter et al., 1993). Although the protective effects of another variant, e2, were investigated and identified as protective against AD subsequently in 1994, the emphasis remained on e4 (E3 can be considered neutral for the purposes of this review). Research on the APOE e2 allele lags behind research on the AD risk allele APOE e4. As a crude metric derived from PubMed (5 December 2021) a search (search terms APOE e2 and APOE e4) found 997 articles relating to e2 and 334 on e4 for the years 1994–2010. In the 2011–2021 period 709 e2 articles and 3,382 e4 articles were published, suggesting diminishing interest in e2 vis a vis e4 over time. Thus, there has been disproportionate work done with a focus on e4. This situation is puzzling because e2’s protective effects against AD are large and if understood could offer therapeutic windows for AD prevention or amelioration. Several excellent reviews provide insight on how differences in APOE allele may confer risk for neurodegeneration or alternatively neuroprotection. These have generally focused on the e4 variant, evolutionary dynamics of APOE, APOE in transgenic (tg) mice, or risk mitigation due to gender or ethnicity (Mahley and Huang, 2012; Mahley, 2016; Belloy et al., 2019). A review of molecular properties of e2 resulting in pleiotropic effects has been published (Li et al., 2020), while a single older review (Suri et al., 2013) examined neuroimaging associations. Hyman and colleagues (Serrano-Pozo et al., 2021) have offered multiple insights into potential biological pathways by which neuroprotection or e4 associated neurodegeneration might occur. In this review, we examine several features of the APOE e2 allele that have not been qualitatively and critically synthesized. These include its associations with longevity, cognition, neuroimaging, biomarkers, and neuropathology, all in humans. We hope to provide some basic parameters that might set the stage for a unified theory of e2 across multiple explanatory levels and provide relevant biomarkers for understanding what a neuroprotective strategy might look like, as well as its limits. We examine molecular mechanisms only schematically, as these have recently been extensively examined (Shinohara et al., 2020; Serrano-Pozo et al., 2021).

### Allele Frequencies and Early Association Studies

Allele frequency for e2 varies very slightly among ethnic groups. In North Americans of European extraction, the frequency of the e2 allele is about 8–10%, the frequency of the e4 allele 15–20%, and the e3 allele frequency is approximately 70%. For Africans and African Americans the allele frequency of e2 is about 11–12% (Murrell et al., 2006). E2 allele frequency may differ among various Hispanic subpopulations with frequencies in the 3–15% range (Gonzalez et al., 2018). In East Asian Han Chinese the frequency is about 12% (Liu et al., 2014).

In 1993, Roses and colleagues (Corder et al., 1993; Strittmatter et al., 1993) demonstrated a strong association between the e4 allele and clinically diagnosed AD (note that the three AD variants were already known). Corder et al. (1994) were the first to observe that in contrast to the “neutral” e3 allele, the e2 allele had a protective effect against clinically diagnosed AD. Farrer et al. (1997) fully confirmed these findings in a US multi-ethnic population of Caucasians, African-Americans, Japanese, and Hispanics. Meta- analytic approaches found that a single copy of the e4 allele has an odds ratio (OR) = 3.6 for clinically diagnosed AD and two copies increased risk with an OR of 12–15. For the e2 allele the OR was 0.54 (Bertram et al., 2007) (see below for some qualifications depending on e2 genotype). E2 appears to be protective in African Americans, where it reduced the OR to a similar degree as in Caucasian samples (Murrell et al., 2006). A recent study suggested that e2 effects may be attenuated but still present in a Caribbean Hispanic population (Blue et al., 2019).

Several other variants have been reported to confer neuroprotection against AD. A mutation in the APP gene in an Icelandic population and the Christchurch mutation in APOE e3 are among very rare protective variants that are relatively established. These will not be discussed in this review.

### APOE Molecular Genetics

**FIGURE 1:**
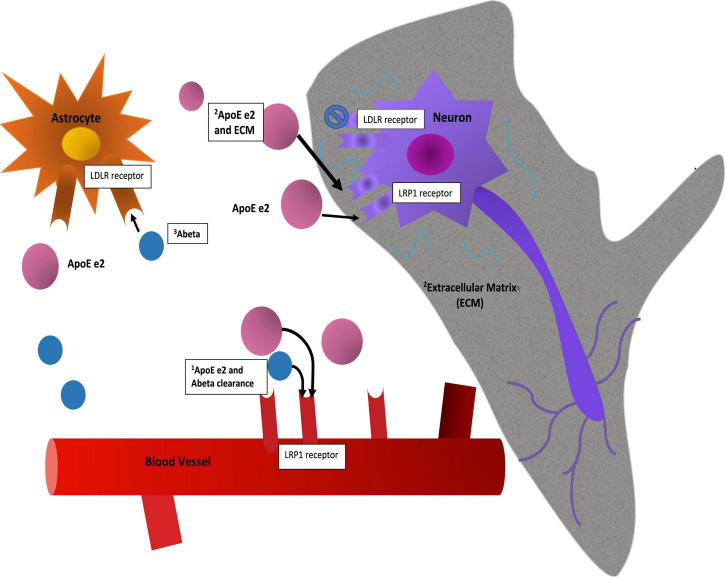
Mechanisms. This figure is a speculative but neurobiologically plausible mechanistic model of how two established molecular properties of the e2 isoform, namely its protein abundance and its low affinity for the LDLR receptor, might provide initial stages of neuroprotection. While mRNA levels of the isoforms appear to be equivalent (Conejero-Goldberg et al., 2014), post translational differences due to isoform related susceptibility to cleavage or other degradation related processes result in full-length protein level differences (Riddell et al., 2008; Mahley, 2016). The mechanistic interpretation of such differences in brain are not established: Speculatively, protein abundance may have advantageous effects in and of itself by way of clearance of Ab, including via the BBB, or delivery of cholesterol to neurons for synaptic maintenance. As a caveat to this, the e4 isoform may be “toxic,” so simply increasing abundance might not be advantageous. Second, neuroprotective effects may be associated with reduced binding at a primary cellular ApoE receptor, LDLR, with the corollary that more ApoE e2 is more available at other receptors, such as LRP1 or ApoER2 and in parallel, allow other ligands to stimulate the LDLR receptor. These and other upstream (e.g., promoter variants) and downstream factors (e.g., isoform specific differences in lipidation, microglial activation, LTP reduction, etc.) are discussed elsewhere.

The APOE gene is triallelic (Strittmatter et al., 1993; Mahley, 2016; Belloy et al., 2019) due to amino acid substitutions resulting from two non-synonymous single nucleotide polymorphisms (SNPs) in its exon 4. E2 has two cysteines at amino acid residues 112 and 158; e4 two arginines, and e3 a cysteine at 112 and an arginine at 158. APOE’s product, ApoE, is the major lipid transporter in brain. Delivery of cholesterol and other phospholipids are advantageous for cell membrane repair and synaptic modeling functions. Full length ApoE protein is distinguished by two domains and a hinge region between them. The C-terminal domain contains the lipid binding region and the N-terminal domain contains the receptor binding region. ApoE is expressed in astrocytes and microglia and may be expressed in neurons when under stress or during aging, though this is considered controversial (Mahley and Huang, 2012). ApoE binds to several receptors including LDLR, LRP1, HSPG, VLDLR, and ApoER2 (Bu, 2009). Binding to the LDLR receptor varies significantly between ApoE e2 and the other isoforms, as e2 binds with very low affinity to this receptor (Ruiz et al., 2005). Furthermore, differences in lipid binding and amyloid Beta protein (Aβ) interaction in the C terminal domain have been observed among the isoforms (Verghese et al., 2013; Li et al., 2020; Serrano-Pozo et al., 2021). Currently, e2 is thought to be hyperlipidated (Yamazaki et al., 2019). There is an on-going debate about the differential mechanisms of ApoE isoform interactions with Aβ, namely whether they are direct or indirect (Serrano-Pozo et al., 2021). Additionally, and due perhaps to differences in conformation of the full-length protein, the e2 isoform is less susceptible to post-translational cleavage or degradation than e3 or e4 (the latter being most susceptible due to access to a hinge region) (Mahley, 2016). These differences result in large differences in ApoE protein abundance and can be observed in brain and in blood in humans, *in vitro* cellular models, and targeted replacement (TR) human APOE mice (Riddell et al., 2008; Bales et al., 2009; Conejero-Goldberg et al., 2014; Rasmussen, 2016). In Figure 1 we show schematically how these two established properties of the e2 isoform, high protein abundance and low affinity for the LDLR receptor, might be mechanisms that promote neuroprotection directly and indirectly.

In addition to post translational isoform specific differences in cleavage and degradation, sialylation differences may be present. Moon et al. (2022) found that e2 had the greatest sialic acid post translational modification, resulting in reduced interactions with Aβ and reduced Aβ pathogenesis.

This review will focus on e2 associations beyond the molecular level. We will provide selective reviews of the e2 associations with (1) longevity; (2) AD neuropathology, tauopathies, and other FTLDs, and CVD related tissue and vessel pathologies; (3) plasma ApoE level as a potential biomarker; (4) cognition and neuroimaging; and (5) non-neurologic pleiotropy. We believe this is warranted because the literature on these topics is often found in disparate journals and disciplines and has neither been qualitatively and critically synthesized, nor focused on humans. Our review is selective as we give prominence to older, seminal studies and recent studies. Our search strategies relied on our own knowledge, targeted PubMed searches, and references from reviewed studies.

## Methods

Articles relevant to the association between APOE e2 and neuroprotection were identified by an electronic database search using PubMed, MEDLINE, and PsycINFO that was conducted from July 2020 to September 2021. These searches were limited to English language and publications after 1994, when e2 was first identified as a genetic factor contributing to AD pathology. Abstracts of identified publications were reviewed, but articles were excluded from our examination if APOE e2 was not mentioned. In addition to the electronic database, we identified additional articles from the reference lists of the studies we reviewed. Information from review papers and meta-analysis papers on APOE and APOE e2 were used to gather additional references or to supplement findings for the current review.

## Longevity

Longevity broadly represents overall health, thus, understanding e2’s potential impact on longevity could provide knowledge on the extent to which e2 impacts neuroprotection as well as overall physiologic robustness. Strong evidence for an e2 effect on longevity comes from examination of centenarians and individuals who are more than 90 years of age. In the first candidate gene study of APOE e2 and longevity that was conducted in 1994 in centenarians, e2 carriers were over-represented with an allele frequency of 12.8% compared to 6.8% in the non-centenarian control group. Conversely e4 was under-represented (5.2 vs. 11.2%) (Schachter et al., 1994). This pattern has been identified in multiple other studies. Several candidate gene studies have been conducted by using a variety of designs (Ewbank, 2004; Sebastiani et al., 2019). The control group generally consists of individuals who are older but have not reached 90 years. As not all were deceased, it is possible that some of these individuals might be long-lived and so reduce power to detect effects on mortality. Perhaps the greater criticism is the use of candidate gene approaches which are subject to the “winners curse” phenomena. Other studies have involved adults with a long-lived parent as a surrogate for longevity; these may introduce noise and reduce power. Nevertheless, the majority of studies have found e2 to be significantly enriched in long-lived individuals (and e4 to be depleted). Indeed, an early GWAS meta-analysis of long-lived individuals identified e2 as a longevity variant (Broer et al., 2015). Studies in South American (Murrell et al., 2006) and Asian populations (Bales et al., 2009) have inconsistently replicated these results but sample sizes have been small and when studies when meta-analyzed, findings were heterogeneous.

The recent meta-analysis of Deelen et al. (2021) addressed many of the design issues highlighted above. Using cases who survived to the 90 or 99th percentile survival based on life tables (11,262 and 3,484) and controls (25,483 and 5,000) who died or had last contact at or below the 60th percentile for survival, and GWAS data with imputation for 73,000,000 SNPs, APOE e2 was found to have the largest association with longevity in the 99th percentile (*OR* = 1.47 discovery and *OR* = 1.35 validation samples) and was highly significant. Results were similar for the 90th percentile for survival, and e2 was the only variant associated significantly with longevity in all samples. Effects did not differ by ethnic group. While the age gap comparisons utilized in the study have been criticized (here between individuals who survived to the 90th or 99th percentile in life tables vs. those who died or whose last contact was prior to the 60th percentile), they may also be considered conservative as some younger individuals may go on to very old age and thus reduce power. In short, these results incisively established e2 as a longevity variant. It is unclear from this and other studies how many years on average e2 adds to the lifespan. One potential explanation for these results is the reduction in AD in which life expectancy is shortened. In examining this hypothesis in e2 carriers, Shinohara et al. (2020) demonstrated that e2 cases had lower hazard ratios (HRs) for mortality (i.e., longer lives) than did e3 homozygotes and e4 cases after statistically controlling for AD pathology. Moreover, in a subgroup analysis of cases with low levels of amyloid plaque, e2 cases again had lower HRs. Interestingly, Olah et al. (2018) also demonstrated that in a gene dataset examining microglia and advanced aging, gene expression was reduced in e2 carriers. These results, among the few in the literature, are important because they suggest that e2 effects on longevity are at least partially independent of its effects on AD. If replicated and mechanistically understood, the results could offer a window for longevity strategies.

## Neuropathology

There are several reasons to examine e2 associations with neuropathology. First, by using gold standard histopathological diagnostics, more accurate estimates of e2 neuroprotective effects might be derived without the vagaries of clinical diagnosis. Second, and conceptually, it allows one to examine if e2 might have general protective effects against multiple late-life proteinopathies with implications both for disease and e2 insofar as neurodegenerative disorders may share common molecular features, including aggregations, that might also be attenuated by common molecular pathways driven by the e2 isoform.

### AD

**FIGURE 2:**
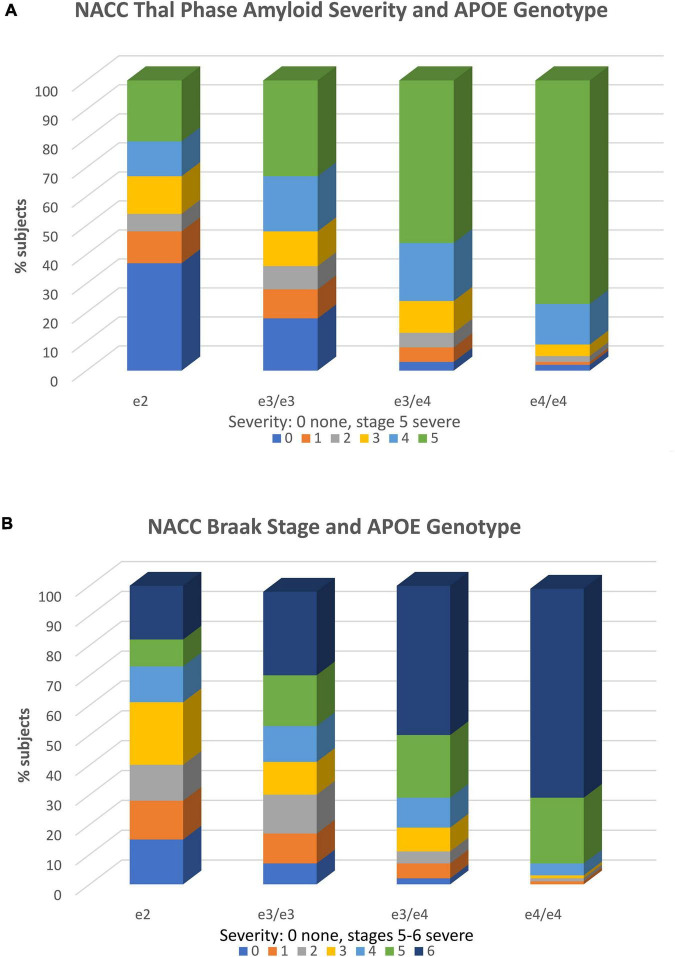
APOE genotype and neuropathology. **(A)** Association of APOE genotype and diffuse amyloid plaque extent (Thal phase). Within each APOE genotype column, colored rows represent the relative proportion of cases in each severity stage. These proportions are expressed as percentages and add to 100. There are increasing proportions of the most severe pathology (plaque stage 5) from the e2 to e4/e4 genotype groups in stepwise fashion. Stage 1 includes one or more neocortical regions with Ab immunopositivity, stage 2 includes hippocampal positivity, and stages 3–5 include other limbic and subcortical structures. Amyloid stage 0 = no pathology; stage 5 = widespread cortical, limbic, and subcortical pathology. Stages 1 and above are consistent with AD. The total *N* = 1,557. Reproduced with permission Goldberg et al. (2020a). **(B)** Association of APOE genotype and Braak stage. Within each APOE genotype column, colored rows represent the relative proportion of cases in each severity stage. These proportions are expressed as percentages and add to 100. There are increasing proportions of the most severe pathology (Braak stages 5 and 6 from the e2 to e4/e4 genotype groups in stepwise fashion. Braak stage characterizes the spread of neurofibrillary tangle pathology from entorhinal cortex (stages 1 and 2), to increasing involvement of the hippocampus (stages 3 and 4), to neocortical involvement (stages 5 and 6) (Braak stage 0 = no pathology; stage 6 = severe neocortical pathology. Stages 3 and above are consistent with AD. The total *N* = 1,557. Reproduced with permission Goldberg et al. (2020a).

The importance of examining APOE e2 associations with neuropathology is evident, as it allows investigators to bypass potential inaccuracies in clinical diagnosis and directly examine established AD histopathologies (clinical diagnostic accuracy typically ranges from 60 to 80% compared to gold standard neuropathological diagnosis). Recently, using this approach, we examined the association of e2 with various tissue neurodegenerative pathologies and blood vessel pathologies in the updated NACC v. 10 Neuropathology Database (Goldberg et al., 2020a). In samples of over 1,500 brains, we found that a combined group of e2/e2 and e2/e3 cases, when contrasted with e3 homozygotes, demonstrated ORs of 0.43, 0.54, and 0.55, respectively and thus significantly reduced risk (*p* < 0.001) for Thal amyloid extent, neuritic plaque density, and Braak stage, all of which are neuropathological indicators of AD. As expected, ORs for e2 vs. combined e3/e4 and e4/e4 carriers were even lower (*OR* = 0.11–0.14). These genotype-pathological associations are shown in Figures 2A,B. There is clear stepwise progression from disproportionate severe levels of pathology in e4/e4 carriers to sharp reductions in the e2/e3/e2/e2 group, along with a concomitant increase in the latter carriers with no or mild pathology.

In a mediation analysis, e2 had both direct and indirect effects on tau Braak stage. The indirect effect was via amyloid neuritic plaque burden and was larger than the direct effect. The direct effect may be more precisely termed a non-amyloidogenic path. Thus, e2 might modulate spread of tangles through two or more processes: indirectly through its effects on Aβ reduction, directly by reducing tau fibrilization, or indirectly through other unknown pathways.

In a neuropathology study that included a large set of e2/e2 homozygotes, Reiman et al. (2020) also found that pathologically confirmed AD cases were significantly reduced when contrasted with e2/e3. In fact, in the neuropathologically confirmed group of 5,007 cases, the e2/e2 genotype displayed an OR of 0.13 and e2/e3 an OR of 0.39 when contrasted with e3 homozygotes. The respective ORs were weaker in the clinically diagnosed group (i.e., e2/e2 = 0.52 and e2/e3 *OR* = 0.63). Results for e2 effects on neuritic plaques and Braak stage were also significant in a Consortium to Establish a Registry for Alzheimer’s Disease (CERAD) series in the Reiman et al.’s study; ORs were not analyzed. Reiman et al. found residual effects for e2 on tau pathology after adjustment for amyloid pathology, broadly consistent with effects Goldberg et al. (2020a) observed in mediation analysis.

In an earlier study, Serrano-Pozo et al. (2015) examined a smaller version (*N* = 792) of the NACC neuropathology database. They found that e2 was significantly associated with reductions in Braak stage, but not neuritic plaque severity (Thal stage was not examined). Consistent with Goldberg et al. (2020a) they also found significant direct and indirect effects of e2 on Braak stage in mediation analyses.

Comparatively few studies have explored the role of APOE e2 in relation to brains which meet criteria for histopathological AD; yet, overall, the results of these studies suggest that e2 is neuroprotective. AD patients that carry e2 are found to exhibit significantly reduced Aβ deposition in the neocortex (Nagy et al., 1995). In addition, e2 carrier AD brains appeared to express less neurofibrillary tangles (NFT) formation (Morris et al., 1995). However other studies demonstrate the opposite outcome in the oldest old with intact cognition (Berlau et al., 2009). In the latter study e2 carriers had more AD pathology than did e3 homozygotes, but were more likely to have preserved cognition, suggesting that e2 provides cognitive or neurobiological reserve. It is possible that the way e2 is manifested in this extreme age group may be more complex, as it might interact with other variables, such as age, longevity, sex, etc.

### The Special Case of e2/e4

The study of e2/e4 genotypes (comparatively rare) may offer insight into understanding the extent of e2 protective effects when the e2 and e4 isoforms are in the same brain. Does e2 neuroprotection win? Does e4 toxicity win? Is there some type of balance? It might also have bearing on clinical studies delivering the e2 isoform to e4 carriers. In a study of e2/e4 carriers in post-mortem cases (*n* = 1,100), these cases showed threefold increased odds of pathologic AD and a higher Aβ load than e3/e3, though associations with tau tangle density were non-significant (Oveisgharan et al., 2018). Braak stage was not examined. Rather unexpectedly, a difference in the risk of clinical AD for e2/e4 compared to e3/e3 (HR = 1.5) was not observed, suggesting that clinical diagnosis can be rather imprecise. Nevertheless, HR of 1.87 for e4 for AD risk did not differ from that of e2/e4. Goldberg et al. (2020a) found that in cases with e2/e4 genotype, risk for AD plaque and Braak stage pathology was similar to e3/e4, not e3/e3 or e2/e3. Copy number was controlled, increasing the rigor of the approach. These results indicate that the e4 isoform’s effects were neither blunted nor otherwise modified by e2 within the same brain, at least insofar as levels of the isoforms are in physiological range. However, in the study by Reiman et al. (2020), e2/e4 had an OR of 2.68, somewhat lower than the OR of 6.13 for e3/e4. With respect to the latter, the results from studies of Oveisgharan et al. (2018) and Goldberg et al. (2020a) (see below) are perhaps more similar to each other than the results from the study of Reiman et al. Nevertheless, all these studies make the observation that e2 does not offer neuroprotection when the two isoforms (e2 and e4) are coexistent in same brain, as risk was increased when contrasted with e3 homozygotes. Results also imply that the pathways by which e2 confers neuroprotection do not overlap with or otherwise blunt those by which e4 confers risk.

### Fronto Temporal Lobe Dementia and Tauopathies

Remarkably, e2 has been shown to promote risk of certain frontotemporal lobar degeneration (FTLD) and tauopathy diseases when neuropathologically defined. In the aforementioned study, Goldberg et al. (2020a) found that e2 promoted pathology in TDP-43, Pick’s disease, and progressive supranuclear palsy (PSP) at the trend level by chi-square (*p* < 0.01), although these became non-significant when AD pathology (defined by ABC neuropathological criteria) was controlled. It is possible that the relationship between FTLD or tauopathies and AD histopathology is complex, or alternatively, that the statistical manipulation introduced artifact. Recently, Zhao et al. (2018) showed that in humans, the e2 allele was associated with increased tau pathology in the brains of PSP cases (with an association between the e2/e2 genotype and risk of tauopathies) using two series of pathologically confirmed cases of PSP and corticobasal degeneration. Sample sizes were large and measures of pathological tau were conducted using both animal models and human brain samples. Several early studies of fronto-temporal dementia (FTD) and/or tauopathies, namely PSP and corticobasal degeneration (CBD), in which pathology was confirmed, have indeed found e2 or e2 homozygotes to increase risk (Verpillat et al., 2002; Rubino et al., 2013; Chio et al., 2016). With a newly identified diagnostic entity, primary age-related tauopathy, e2 (and e4) has been found to be over-represented in a pathological series comprised largely of cases with preserved cognition and reminiscent of findings in FTLD and related tauopathies (Robinson et al., 2020).

Taken together, these results demonstrate how specific the protective effects of e2 are (e.g., they seem specific to tau 3R/4R found in AD and prevent tangles); it may also be the case that despite some evidence for a direct effect on tau, most of e2’s protective associations with tau in AD are via amyloid mediation, but this would not explain e2 as a risk allele. In sum, e2 is protective against AD-related neuropathologies, such as Aβ and tau Braak stage, but not other proteinopathies. Remarkably, e2 may promote some tauopathies and FTLDs.

### CVD

In a focused study of cerebral amyloid angiopathy (CAA) in post-mortem samples, Nelson et al. (2013) found that e2 cases had a significantly higher proportion of CAA than did e3/e3 cases, and this increase in frequency was similar to that of e3/e4 cases. While multiple MRI studies have examined APOE genotype associations with lobar hemorrhage (with both e2 and e4 increasing risk) relatively few neuropathology studies have been conducted. Lobar hemorrhage associations with e2 have been observed. Goldberg et al. (2020b) examined the associations of e2 with CVD in the NACC v10 database. They did not find that e2 was associated with protection from infarcts, microinfarcts, and microhemorrhages when contrasted with e3/e3 genotypes. In the imaging section described below, e2 appeared to increase risk for WMH, an MR indicator of small vessel disease. E2 was, however, significantly associated with increased risk of gross hemorrhage (including lobar hemorrhage), notably in the presence of CAA. As CAA was present in about 50% of e2 cases, this increased risk was not infrequent. Gross hemorrhage may correspond in part or wholly to intracerebral hemorrhage; the latter has been found to be associated with e2 in other studies (see neuroimaging section). In the Goldberg study (Goldberg et al., 2020b), e2 cases also had a higher proportion of CAA than did e3 homozygotes.

### Summary

In summary, e2/e2 and e2/e3 have highly significant associations with reduced Aβ and tau pathologies that are consistent with AD, with two copies demonstrating larger effects than one copy of e2. This work also yields three cautionary points:

1. Considering the risk associated with e2/e4, introducing e2 in the presence of e4 at physiological levels may not reduce the risk of some neurodegenerative disorders.

2. e2 does not have an equivalent effect on multiple proteinopathies and it may sometimes promote a pathology (see for example Goldberg et al., 2020b on tauopathies) and, moreover, does not offer protection even when the protein constituents are similar to those on which it has an effect, namely 3R and 4R species of tau.

3. e2 increases risk for an Aβ related vessel pathology, CAA, and when CAA is present, increases risk for lobar hemorrhage.

## Plasma ApoE and Dementia Risk

Plasma ApoE levels, insofar as they are surrogates for brain protein levels, may yield information as to APOE e2 mechanisms and/or serve as a biomarker with utility of its own. Plasma and serum ApoE levels follow an isoform specific profile such that ApoE is most abundant in e2 carriers, compared to other alleles (e2 > e3 > e4) (Soares et al., 2012). This difference is thought to result from the post translational stability of the e2 isoform (i.e., lower propensity for cleavage in the hinge region of the protein or degradation).

Although ApoE in plasma and brain derive from different sources (liver and astrocytes, respectively), plasma ApoE can in principle be used as a proxy for brain ApoE for the following reasons: 1. It demonstrates the same stepwise association with APOE genotype as it does in brain (Bales et al., 1997; Soares et al., 2012; Conejero-Goldberg et al., 2014; Rasmussen, 2016); 2. It is directly correlated with cortical ApoE levels (e.g., hippocampus ApoE and plasma ApoE *r* = 0.78) in TR mice (Shinohara et al., 2013); and 3. Shows inverse relationships with AD biomarkers (Conejero-Goldberg et al., 2014; Apostolova et al., 2015) as does brain ApoE in TR mice (Shinohara et al., 2016a).

In a population study of over 75,000 Danes, Rasmussen and Frikke-Schmidt examined plasma ApoE levels and risk of dementia (Rasmussen et al., 2015). They found that 1. Both APOE genotype and plasma level were strongly associated with risk of dementia (such that e2 had the highest levels and e4/e4 the lowest levels) and genotype accounted for about 0.25 of the variance in plasma ApoE, suggesting that ApoE levels are partially independent of genotype 2 (Rasmussen et al., 2015). In univariate analyses, both genotype and plasma levels had a highly significant impact on AD dementia risk, with e2 and high level of plasma ApoE being associated with lower risk; and 3. No interaction between genotype and plasma APOE on dementia risk. Promoter and rare exonic variants also accounted for some of the differences in ApoE level (Rasmussen et al., 2015).

ApoE protein abundance has been examined in non-AD post-mortem human brain by immunoassay in a study by Conejero-Goldberg et al. (2011). They found a stepwise pattern in protein abundance among isoforms such that e2 > e3 > e4. However, the number of cases per group was small as in earlier studies (see also Beffert and Poirier, 1996). Nevertheless, this replicates the findings that APOE TR mice demonstrated reliable differences among the isoforms with this profile.

Several studies have directly examined plasma ApoE level effects on cognitive markers and biomarkers. Over a restricted age range, Teng et al. (2015) found that lower levels of plasma ApoE were associated with greater hippocampal atrophy in the ADNI dataset. Low levels of plasma ApoE have been consistently correlated with PiB PET Aβ positivity independent of APOE genotype (Kiddle et al., 2012; Apostolova et al., 2015; Gupta et al., 2015; Lazaris et al., 2015). Yasuno et al. (2012) found a significant, similar relationship between plasma ApoE and cognition in a large older healthy control sample with a medium effect size. In all these studies, higher levels of ApoE were associated with better outcomes. A recent study of symptom resilience in ADNI using advanced LASSO regression approaches found that increased ApoE protein (along with microglial activation and chemotaxis) were predictive of better cognitive outcomes over 4 years in ADNI’s healthy controls, MCI, and AD individuals in both training and validation samples (Meyer et al., 2019). ApoE level was the most consistent and robust predictor.

An earlier literature not summarized here is less consistent (e.g., Taddei et al., 1997; Thambisetty et al., 2011; Martinez-Morillo et al., 2014). Additionally, mass spectrometry assays have generally not found isoform related differences in ApoE for unclear reasons (Thambisetty et al., 2011). Newer literature with larger better characterized samples (i.e., ADNI and AIBL) has yielded more consistent results. However, most studies have assayed total plasma ApoE and have not measured ApoE in an isoform specific manner in APOE heterozygous individuals. Thus, studies are not as informative as they might be.

Conversely, it should not be overlooked that APOE genotype may have effects independent of plasma ApoE level because genotypes may have important biological properties unrelated to the protein level. Such properties include isoform conformation that (1) impacts binding to the LDLR receptor such that the e2 isoform has very low affinity potentially increasing the Aβ or tau uptake at this receptor; (2) reduced cleavage products that have presumptive toxicity due to impact on mitochondria (Mahley and Huang, 2012); and (3) lipidation status impacting synaptic maintenance, arguably reduced in e4 and increased in e2 individuals (Serrano-Pozo et al., 2021).

## Cognition

In principle, important information about when and if e2 exerts an effect on cognition can be gleaned from studies in which age range was delimited and various cognitive domains or constructs were observed to examine what might be most sensitive to modulation by e2 genotype. Moreover, cognitive impairment or decline is the most prominent clinical symptom of AD and is the key target for treatment. It is therefore crucial to thoroughly and critically examine the role of e2 in various cognitive domains. We identified 14 cross-sectional and 22 longitudinal studies on the association between APOE e2 and cognition and our findings are summarized in Supplementary Table 2.

A small number of studies examined the effect of APOE e2 on cognition in young adulthood and midlife, and these studies were restricted to cross-sectional findings. In a study by Sinclair et al. (2017), a trend for e2 to be associated with better episodic memory was noted in young adults (aged 22–23 years), and better performances on processing speed and executive function tasks were reported in both young and middle-aged adults (aged 42–67 years) who were e2 carriers. Two experimental studies reported disadvantageous or comparable performance in e2 carriers on visual search paradigms, though these studies were limited by small sample size (*n* < 20) (Greenwood et al., 2000; Lancaster et al., 2017).

It is unclear if e2 has a protective role on cognition of older adults. Older adults who are e2 carriers performed significantly better on cognitive domains pertaining to the frontal network (e.g., processing speed, attention, and executive functions) in some studies (Sinclair et al., 2017), while the majority of findings did not find any significant associations with e2 or even demonstrated adverse effects on cognitive performance (Greenwood et al., 2000; Bennett et al., 2009; Alfred et al., 2014; Marioni et al., 2016; Palmer Allred et al., 2016; Lancaster et al., 2017). Verbal fluency (phonemic and categorical), language, and visual-spatial functioning were not significantly associated with e2 in all studies that examined these domains. Findings on the cross-sectional associations of e2 with global cognition and episodic memories have also been mixed, regardless of methodology, sample (cognitively health or MCI/AD), and comparison groups (e3 homozygotes or mix of e3 and e4 genotypes).

Findings from longitudinal studies have produced inconsistent conclusions about the role of e2 on cognitive decline, though a slightly more robust effect of e2 was noted in global cognition (10 out of 15 studies) and memory (7 out of 15 studies). Indeed, in a large and very well-conducted study in a female religious order group (>65 years old), e2 carriers demonstrated very large and advantageous differences in rates of decline in episodic memory but not in other domains over an 8-year period (Wilson et al., 2002). A longitudinal study from the Danish 1905 Birth Cohort with three follow-up visits demonstrated that the protective effect of e2 on global cognition was strengthened across study waves, indicating that the positive effect of e2 against cognitive decline may increase over time (Lindahl-Jacobsen et al., 2013). Nonetheless, further studies are needed to confirm this finding, given that studies from the Lothian Birth Cohort suggest that there may be stronger cross-sectional relationships between e2 and cognitive performance compared with longitudinal relationship (Deary et al., 2004; Schiepers et al., 2012). In no study were e2 carriers significantly worse than other APOE genotypes.

It is also postulated that the effect of e2 may be more prominent in old-older adults (≥75) (Staehelin et al., 1999; Rajan et al., 2019) and in women (Hyman et al., 1996; Davies et al., 2014; Shinohara et al., 2016b). Whether there is a racial difference in the effect of e2 is inconclusive, as only three studies (Blair et al., 2005; Palmer Allred et al., 2016; Rajan et al., 2019) included ethnically diverse samples and only one found a significantly smaller effect in African-Americans compared to Caucasian Americans (Blair et al., 2005).

Clinical variables may play important moderating roles in the relationship between APOE genotype and cognition. E2 may function through different mechanisms in various AD stages (e.g., MCI and AD), and understanding how e2 interacts with AD diagnosis is imperative for understanding its role in pathologic brain aging. Studies that examined e2’s role across AD stages produced mixed findings, with some suggesting e2 protected against cognitive decline only in cognitively healthy older adults (Blacker et al., 2007), and some implying that e2 was associated with slowed cognitive decline in AD (Martins et al., 2005; Serrano-Pozo et al., 2021). Notably, Conejero-Goldberg et al. (2014) found that in ADNI data that MCI e4 carriers had a greater progression rate to AD, compared with e2 (HR for e4 vs. e2 was 3.95). E2 may interact with the neurodegeneration process to impact cognition, as a study by Gong et al. (2019) found that the hippocampal volume and thalamus function mediated e2-associated cognitive performance in MCI, but not healthy older adults. These findings suggest that disease states could modify the APOE-cognition interaction, but more studies are warranted on this topic.

Recent evidence from imaging and neuropathology biomarkers provides a current consensus that individuals with e2/e4 “behave” more closely to those with e4, leading to advanced AD pathology, and these findings are consistent with e2 studies on cognition. One of the earliest studies that explored the relationship between e2/e4 genotype observed a superior cognitive performance in the rare e2/e4 group among e2 carriers, but this study was limited by a small sample size with the majority of the e2/e4 group (11 out of 16) belonging to the “young-old” group (<75 years old) (Staehelin et al., 1999). Recently, a study by Rajan et al. (2019) studied differences in cognitive performance by different e2 genotypes (e2e2 vs. e2/e3 vs. e2/e4) and reported relatively poor performances among individuals with e2/e4. Other studies have also demonstrated that individuals with e2/e4 genotype performed at the level similar to those of e4 homozygotes (Greenwood et al., 2005; Oveisgharan et al., 2018), in addition to e3 homozygotes and other e2 groups (e2/e2, e2/e3) (Oveisgharan et al., 2018). The rate of decline for e2/e4 was similar to both e3/e4 and e4/e4. Similarly, some findings (Shinohara et al., 2016a) suggested protective effects of homozygous e2 alleles but due to its extremely low prevalence, further studies with large-scale datasets are needed to strengthen the current understanding of e2 homozygotes on brain function.

### Conclusion

Studying the relationship between APOE e2 and cognition is complex, and findings are largely mixed due to different study samples, small number of e2 carriers, and different methodologies. About half of the studies reviewed on this topic (17 out of 36 studies) found no effect of APOE e2 on any cognitive domain, providing insufficient evidence for the e2 allele’s suggested effect of protection against cognitive impairment/decline. It would make conceptual sense if there were strong evidence of e2 protective effects in later life longitudinally, this was not always the case. Nevertheless, perhaps the strongest study based on multiple cognitive domains, length of follow up, and sample size (Wilson et al., 2002) found strikingly less memory decline in the e2 group contrasted with e3 homozygotes. Given that there are also substantial null findings between e2 and memory, their relationships should be explored further in various samples. Studies that found significant association between e2 and cognitive protection, broadly defined, tended to focus on older adults, aged >75 years. Nonetheless, the small number of existing longitudinal studies and a lack of long-term follow-up are major limitations on this topic, and we are currently unable to discern whether cognitive performance is significantly and independently associated with e2. Interestingly, e2 may have a significant role in cognition across different disease states (e.g., MCI and AD). Given that neuroimaging findings suggest the presence of interaction between clinical status and neuroprotection, examining whether similar patterns exist with cognition will be crucial to understand the role of e2 in various stages of AD and pre-AD.

Prominent methodological discrepancies across studies were noted in the definition of the “e2 groups” and inclusion/exclusion of e2/e4. Given that e2/e4 may have a distinctive role in brain pathologies and cognitive performance, future studies should investigate this genotype separately from other e2 allele groups. The definition of reference groups (e.g., e3/e3 or any non-e2 carriers) also varies somewhat across studies, although findings were still inconsistent even among studies that rigorously examined e2 against e3 homozygotes. Lastly, restricted range of cognitive domains and lack of diverse samples may also contribute to mixed findings. While some findings indicate that APOE e2 may indirectly effect cognition through mediation of neuropathology biomarkers (e.g., tau and Aβ) (Kantarci et al., 2012), more large-scale studies examining both brain biomarkers and cognition across different stages of clinical diagnosis may clarify APOE e2’s protective effect in cognitive aging.

## Imaging Biomarkers

MRI morphometric measures and functional connectivity might yield important information about the regional impact of e2 or its effect on activation and efficiency during task-based paradigms and network connectivity. Additionally, such data might result in *in vivo* information on microvasculature changes associated with e2. MRI-based measures of neurodegenerative biomarkers have been widely used in quantitatively examining the role of APOE e2 on the brain of both healthy and cognitively impaired individuals. We identified 26 studies (23 cross-sectional and 3 longitudinal) that assessed the impact of the e2 allele on brain morphometrics. Findings are summarized in Supplementary Table 2. The table is sorted by different age groups, in the order of youngest to oldest. The table also summarizes definitions of e2 and its comparison groups. Some studies have notably made comparisons between e2 and combined e3 and e4 groups (e.g., e3/e4, e4/e4), which may not accurately capture the neuroprotective effect of e2 (vs. the neutral e3/e3 homozygotes).

### Cortical Thickness

Cortical thickness or volume, measured in structural MRI, is a common indicator of age-related and neurodegenerative related cortical atrophy. Research has consistently found that APOE4 is associated with lower cortical thickness. However, the relationship between APOE e2 and cortical thickness is a matter of debate. Liu et al. (2010) explored this topic across different AD stages and found that e2 was significantly associated with greater global measures, such as gray matter volume and smaller ventricles, compared with e3 homozygotes or e4 carriers, but this was only true in individuals with MCI or AD and not healthy controls. A study by Serra-Grabulosa et al. (2003) contradicted these findings when examining this topic in older adults with age-related memory impairment, though this study was smaller and did not use standard MCI/AD criteria to define impairment. Longitudinally, one study in individuals with subcortical vascular mild cognitive impairment demonstrated that e2 carriers had slower rate of global atrophy (Kim et al., 2017).

Hippocampal volumetry is a well-established marker of neurodegeneration in AD. Studies in young and middle-aged and older adults have generally shown a tendency for larger hippocampal volume in e2 carriers (den Heijer et al., 2002; Fennema-Notestine et al., 2011; Hostage et al., 2013; Khan et al., 2017; Gong et al., 2019). Findings in older adults are mixed and inconsistent. Large-scale studies (including ADNI, Rotterdam Study, and multi-cohort dataset) indicate that hippocampal volume in e2 is comparable to e3 homozygotes across all clinical and pre-clinical AD stages, though some studies (den Heijer et al., 2002; Blair et al., 2005; Fan et al., 2010; Liu et al., 2010; Fennema-Notestine et al., 2011; Hostage et al., 2013; Conejero-Goldberg et al., 2014; Khan et al., 2017; Groot et al., 2018; Roe et al., 2018) (but not all Liu et al., 2010; Bunce et al., 2012; Gong et al., 2019) suggest that e2 may be associated with larger temporal lobe regions more broadly. Notably, a meticulously conducted 2-year follow-up study from ADNI reported that cognitively normal e2 carriers exhibited a slower rate of hippocampal atrophy, compared to e3 homozygotes, providing strong evidence toward e2’s protective role against hippocampal atrophy (Chiang et al., 2010).

In summary, there are mixed findings on whether APOE e2 has associations with global or regional brain morphometries. There is stronger evidence showing that it may impact the rate of change in brain volume over time, but more replications are needed given the small number of longitudinal studies on this topic. Additionally, findings from MCI and AD populations have been slightly more consistent on the protective role of e2 in brain morphology overall (but less so in the hippocampus). While the proportion of e2 was substantially smaller in these clinical populations, these findings support the notion that the impact of e2 on brain morphology may depend on the stage of the AD pathology. One could argue that e2’s protective role may be more robust later in the course of the disease when it contributes to increased neuroanatomical reserve against cognitive decline; however, there is currently a lack of data on the severity or prognosis of AD pathology within e2 carriers once they develop clinical MCI or AD. It should also be noted that many findings on MCI and AD were from well-characterized but overlapping samples from ADNI, and more investigations from other large-scale studies are warranted to explore this topic furthermore.

### PET Amyloid-β Deposition

Given prior neuropathological findings, we examined whether *in vivo* biomarkers align with gold standard neuropathological associations with APOE e2. Aggregation of cerebral Aβ is among the key biomarkers of AD and is one of the earliest detectable pathologic events in AD progression (Jack et al., 2010). Studies that used positron emission tomography (PET) to assess Aβ pathology in APOE e2 have consistently demonstrated a protective effect of e2 on Aβ accumulation (Morris et al., 2010; Grothe et al., 2017; Lim et al., 2017; Roe et al., 2018). In the progression of AD, e2 appears to be associated with later age at onset of Aβ positivity; for example, a meta-analysis conducted by Jansen et al. (2015) indicated that, compared to e3 homozygotes, the odds of exhibiting Aβ positivity at age 70 was reduced to about 30% in e2 homozygotes, whereas the odds were 18 times higher in e4 homozygotes. Similarly, longitudinal studies jointly pointed to a significantly slower rate of Aβ plaque accumulation in older adults who were healthy at baseline (Hall et al., 2019) and in those with cognitive impairment (Kim et al., 2017).

In conclusion, the strong association between e2 and Aβ aggregation in PET imaging indicate that APOE genotype may be an important consideration for treatment of AD pathology, particularly in relation to amyloidosis. These findings were also strongly represented in e2 vs. e3 comparisons. We could not identify a study that focused on e2 and PET-based tau outcomes, though future studies using both PET amyloid and tau may help clarify the role of e2 on the development of AD biomarkers, particularly based on the amyloid cascade hypothesis (Jack et al., 2010).

### White Matter Hyperintensities

White matter hyperintensities (WMH) seen in MRI usually have vascular origins and are strongly tied to small vessel cerebrovascular risk factors and outcomes. The burden of WMH has been correlated with poorer cognitive function, dementia, and mortality (Prins and Scheltens, 2015; van den Berg et al., 2018). Because e2 has been considered a vascular risk factor, findings on its association with WMH are corroborated by literature on e2 and increased risk for CAA, microbleeds, and lobar intracerebral hemorrhage (Greenberg, 1998; Biffi et al., 2010; Schilling et al., 2013). Biffi et al. (2010) found that e2 had an *OR* = 1.82 for ICH and similar to that of e4 (*OR* = 2.20). In a systematic review and meta-analysis of 9 studies that examined the association between e2 and WMH, e2 was found to be associated with increased WMH burden (Rajan et al., 2019). A more recent Spanish epidemiologic study indicated a protective effect of e2 on WMH burden, but the sample was much younger (ages 51–64) and generally showed very low WMH as expected in this age group, thus making interpretation of results difficult (Salvado et al., 2019). The mechanism behind this association may be related to reduced integrity of amyloid-affected cerebral vasculature (i.e., CAA), but large WMH observed in e2 carriers with subcortical infarcts and leukoencephalopathy (CADASIL; a non-amyloidogenic angiopathy) also indicates that e2 may increase WMH using amyloid-independent pathways (Gesierich et al., 2016). Interestingly, a more recent study by Luo et al. (2017) also observed that e2 carriers had similar levels of WMH compared to e4 carriers but demonstrated that e2 carriers did not exhibit WMH-dependent cognitive impairment. This suggests that e2 carriers may have some form of cerebral reserve.

These findings consistently indicate that APOE e2 does not exhibit a protective effect on WM pathology; rather it may promote cerebrovascular risk. While most studies on this topic found aversive impact of e2 on WMH (Schmidt et al., 1997; Raz et al., 2012; Groot et al., 2018), the lack of correlation between WMH and cognition in e2 carriers observed in a study by Luo et al. (2017) may help explain why there are better clinical AD outcomes with e2 despite increased such abnormalities.

### White Matter Integrity

Diffusor tensor imaging (DTI) is used to detect the earliest AD changes by exploring microstructural myelin related changes or abnormalities in the brain (Weston et al., 2015). Decreased fractional anisotropy (FA) and mean diffusivity (MD), both DTI measures of white matter organization and integrity, have been found to be sensitive to AD-related changes in the white matter (Stebbins and Murphy, 2009). Nonetheless, the contribution of e2 on WM integrity is unclear, due to the limited number of studies on this topic and interpretative difficulties associated with the measures themselves. Interestingly, Westlye et al. (2012) found that e2 had lower WM integrity (as reflected in low FA and high MD), compared to e3 homozygotes, while Chiang et al. (2012) observed a significantly higher FA in multiple brain regions among e2 carriers, compared with e3 homozygotes. Given that the study by Chiang et al. (2012) were significantly older (mean age 68 years vs. 47 in study by Westlye et al. (2012) and that the structure of myelin undergoes dynamic alterations throughout adulthood, further studies in different age groups may reveal age-related differences in how APOE e2 impacts white matter integrity in cognitive aging.

### Functional Imaging

As the field of neuroscience has advanced to understand the integrity of the brain from between- and within network approaches, it is also critical to understand e2’s neuroprotective role with regard to functional connectivity among various brain regions. To the best of our knowledge, 10 studies have investigated the role of APOE e2 on fMRI-based metrics. Interestingly, the majority of these studies observed similar alterations in functional connectivity (FC) in e2 and e4 carriers (relative to e3 homozygotes) particularly in the default mode network (DMN) (Trachtenberg et al., 2012b; Shu et al., 2016; Gong et al., 2017, 2019). Only one study by Gong et al. (2017) indicated more stable FC in amygdala in e2 carriers, compared with e4 carriers. Brain activations in e2 and e4 were also nearly identical during memory and non-memory tasks (Trachtenberg et al., 2012a). Nonetheless, another task-based fMRI study by Mondadori et al. (2007) found similar FC patterns in e2 and e4 carriers during an initial presentation of a memory task, but these patterns were altered in opposite directions with successive trials (increased activity in e2 carriers vs. decreased activity in e4 carriers). Wang et al. (2012) also found that local synchronization of spontaneous resting state fMRI followed different patterns for e2 and e4 carriers during “off-line” memory consolidation, raising the possibility of differential effects of APOE e2 by various stages of learning and memory. Indeed, a decline in hippocampal activation during a memory task was noted in cognitively healthy older adults with APOE e2, compared with those with e4, despite similar performance on the memory tasks (Nichols et al., 2012). Because greater activity could suggest a compensatory response to deteriorating neural mechanisms (Dickerson et al., 2004; Miller et al., 2008), the authors suggested that these findings support the protective role of APOE e2 on brain functions.

Additionally, other studies that showed an advantageous FC profile in e2 carriers suggest that e2 may have protective role in brain network connectivity with increasing age (Shu et al., 2016) and with AD pathology (Chen et al., 2015; Yuan et al., 2016). Of note, e2 carriers with amnestic MCI had increased FC in the DMN of the entorhinal cortex, one of the earliest brain areas impacted by AD (Chen et al., 2015; Yuan et al., 2016).

Overall, fMRI findings on the role of APOE e2 are remarkably inconclusive across the small number of studies. Furthermore, different approaches to FC methods make interpretations of allele comparisons difficult. There are mild indications of advantage of e2 in task-based MRIs and with age and AD progression, but further investigation with a wide range of AD development stages (HC, MCI, and AD) may clarify the role of e2 in age-related neurodegeneration, and functional activity and connectivity. The strong possibility that many interpretations of FC results are *post-hoc*, given the lack of a consensus-based model of e2 associated BOLD response, is also problematic.

### Conclusion

Brain imaging markers provide important neuroanatomical mechanisms behind the development of AD; nonetheless, existing literature on e2 and imaging markers show inconsistent findings, raising more questions about the role of e2 allele on brain structure and functioning. Out of the 24 studies reviewed, 16 studies demonstrated the presence of protective effect in e2 to some degree. Regarding APOE e2’s role in brain morphology, more than half of the studies indicated a greater cortical thickness or volume, particularly in the entorhinal cortex and hippocampus. However, some studies compared e2 against all other genotypes, including previously established risk allele (e4), and it is unclear whether the “protective effect” of e2 is mostly relative to the negative impact of the e4 alleles on the brain. Most studies that compared e2 against e3 homozygotes suggested comparable or somewhat more advantageous effect of APOE e2 on cortical thickness, but more replications are warranted given inconsistent findings across different sample populations and target regions (e.g., hippocampus). The greatest consensus was found in APOE e2’s association with lower level of Aβ and the slower rate of Aβ accumulation. While Aβ_42_ levels in the CSF correlate well with increased Aβ level in the brain PET imaging, amyloidosis measured in both modalities supports the protective role of e2 on Aβ pathology in middle-aged and older adults. Tau PET imaging and newly developed plasma tau assays may help increase the current understanding on APOE e2 and tau accumulation.

While a considerable number of studies supported e2’s protective role in the brain, there were also studies that did not support its association with cortical thickness, DTI, and fMRI outcomes. Therefore, we cannot conclude at this time that e2 exhibits substantial brain-health advantages across brain imaging outcomes, other than potentially Aβ presence and accumulation.

## Cerebrospinal Fluid Biomarkers

Robust support for an amyloid-protective effect of the APOE e2 allele comes from studies examining Aβ_42_ levels in cerebrospinal fluid (CSF). CSF Aβ_42_ reflects the soluble Aβ pool and correlates with amyloid depositions in the brain (Strozyk et al., 2003). Low level of CSF Aβ has been demonstrated as a precursor to Alzheimer’s disease (Jack et al., 2010). While e2’s association with neuropathology and PET Aβ has been more well-established, corroborating findings with CSF Aβ could clarify e2’s association with amyloid formation. Cross-sectional studies have consistently found a higher level of CSF Aβ_42_ in e2 carriers (Morris et al., 2010; Conejero-Goldberg et al., 2014; Toledo et al., 2015; Grothe et al., 2017; Roe et al., 2018), and e2 was also associated with lower frequency of CSF-defined amyloidosis (Hohman et al., 2017). Similarly, a longitudinal study using ADNI data also demonstrated increased CSF Aβ_42_ over 2 years of follow-up among e2 carriers (Chiang et al., 2010). A large cohort of healthy adults across the lifespan also found that e2 carriers presented overall higher values of Aβ_42_ throughout older ages and that e2 has a protective effect among Aβ positive individuals (Toledo et al., 2015).

CSF tau biomarkers are often used as markers of neuronal injury or neurodegeneration (Jack et al., 2016). Three studies that examined the level of total CSF tau (t-tau) in e2 carriers consistently failed to find a protective effect of e2 on t-tau accumulation (Conejero-Goldberg et al., 2014; Toledo et al., 2015; Grothe et al., 2017). Some indications of reduction in phosphorylated tau (p-tau) (Chiang et al., 2010; Fan et al., 2010; Conejero-Goldberg et al., 2014) could suggest that e2 may have a specific role in reducing phosphorylated tau (at threonine 181) (Verghese et al., 2013), but this needs further exploration given that p-tau levels in e2 carriers were comparable to those of e3 homozygotes in several large-scale studies that included large sample of APOE e2 (Morris et al., 2010; Toledo et al., 2015; Grothe et al., 2017). This contrasts with neuropathological studies in which e2 was associated with large and significant reductions in Braak stage. Understanding the inconsistency between e2 and CSF tau associations vs. e2 and neuropathologically characterized tau awaits elucidation.

## APOE e2 Pleiotropy for Risk

Studies of e2 effects on metabolic disorders yield no clear pattern or findings beyond the association of Type III hyperlipoproteinemia and e2 homozygosity (though cases even in such homozygotes are infrequent, i.e., about 5–10%). APOE e2, while having an association with lipoprotein levels and triglycerides (Rasmussen, 2016), does not appear to increase risk for cardiovascular events (Xu et al., 2016). In contrast, APOE e4 increases risk for cardiovascular events (Goldberg et al., 2020a). A recent meta-analysis found that the e2/e3 genotype was not associated with Type 2 diabetes (T2DM), while the association of e2/e2 was significant, perhaps driven by Type III hyperlipoproteinemia. A disorder in which somewhat consistent findings for an association of e2 with risk is age related macular degeneration (ARMD) (Klaver et al., 1998). In a large data set, one resulting from pooled cases and controls (*N* = 21,160), e2/e2, but not e2/e3, was found to increase risk for ARMD (*OR* = 1.83), while e4 carriers demonstrated reduced risk (McKay et al., 2011). Extending these results e2 was found to have an *OR* = 1.40 vs. e3/e3 in a large study conducted in Australia (Adams et al., 2012). However, associations of e2 with risk for ARMD have not been confirmed by meta-analysis (Xiying et al., 2017). Based on these results it is highly unlikely that e2 is protective and it may be risk promoting.

With respect to neuropsychiatric associations a single small study found that e2 promoted risk for post-traumatic stress disorder (PTSD) (Johnson et al., 2019). Further studies were negative as was a meta-analysis (Roby, 2017), suggesting this was an exemplar of the “winner’s curse.” No studies have been published in the area of e2 associations with personality traits. Association studies of e2 and traumatic brain injury outcome and chronic traumatic encephalopathy have not been conducted in adults, though e4 has been associated with poor outcome in the former (McFadyen et al., 2021). To the best of our knowledge no GWAS study of schizophrenia or bipolar disorder has implicated APOE variants as risk-promoting or protective.

## Sex Differences in Neuroprotective Role of APOE2

Studies that focused on APOE4 have concluded that there is an increased risk of AD in female e4 carriers, compared with male counterparts (Farrer et al., 1997; Altmann et al., 2014; Neu et al., 2017). Although less is understood about if APOE e2 has gender specific neuroprotective effects, the few studies that examined sex differences in APOE2 manifestations suggested that, similar to enhanced effect of APOE4, APOE2 may have more robust neuroprotective effect in females. These studies have demonstrated an increased protective effect in female e2 carriers, compared with male e2 carriers (Hyman et al., 1996; Shinohara et al., 2016a; Neu et al., 2017; Lamonja-Vicente et al., 2021). For instance, Neu and colleagues (Neu et al., 2017) found that women with e2/e3 genotype exhibited decreased risk of AD, with OR of 0.51 (CI 0.43–0.61) compared with e3 homozygotes, while this protective effect was less pronounced in males with e2/e3 genotype (OR 0.71, CI 0.60–0.85). A more recent study by Lamonja-Vicente and colleagues (Lamonja-Vicente et al., 2021) found that the advantageous effect of APOE2 on cognitive performance was found in female e2 carriers but not in male counterparts. Neurobiologically, the effect of sex on molecular pathways (i.e., serum metabolites) are observed particularly in APOE2 (Zhao et al., 2020), which may help explain different clinical manifestation in female APOE2 carriers.

## Discussion

The aim of this review article was to identify and summarize the current knowledge on APOE e2 allele, which has been largely understudied in humans despite its implications for AD risk and pathology and therapeutics. Robust and consistent findings on e2 were on its effect on longevity, especially the disproportionate number of e2 carriers in the oldest old. APOE e2’s association with longevity may be also influenced by reduction of AD risk in e2 carriers as e2 carriers are less likely to develop clinical AD, a factor that shortens life expectancy. An important study demonstrated that this increase in longevity may be partially independent of AD pathology (Shinohara et al., 2020).

A second robust finding was that APOE e2 significantly lowers ORs for amyloid distribution, neuritic plaque density, and tau neurofibrillary tangles. E2’s association with PET amyloid imaging and CSF Aβ were consistently strong and protective. Its association with CSF total tau and p-tau was less consistent for unclear reasons, but given e2 effects on post-mortem tangle pathology, the CSF results should be considered inconclusive. It was further suggested that e2 has direct and indirect effects on AD-related Braak stage tangle pathology. A non-amyloidogenic path may be related to e2 interactions with microglia (Shu et al., 2016), or through its effects on the extracellular matrix gene expression in human post mortem cortex (Conejero-Goldberg et al., 2011). Furthermore, ApoE protein abundance in brain and plasma was reliably found to be greater in e2 carriers compared to e3 homozygotes or e4 carriers. Greater plasma levels have been associated with reductions in risk biomarkers and risk for progression to AD.

For FTLD and tauopathies, e2 was *not* protective. The specificity was quite remarkable as e2 is protective against mixed 3R and 4R species of tau in AD, but not predominantly 3R or 4R tau in various FTLD and tauopathies (e.g., 3R in Picks and 4R in PSP) (albeit the latter tauopathies may have specific cell type predilection or morphology that set them apart from AD tau species). For CVD related MRI and neuropathological findings in tissue and vessel walls, e2 was not protective and in some instances may have promoted disease, as e2 appeared to increase the risk of blood vessel pathology, including CAA, CVD related tissue pathology, namely intracerebral hemorrhage, and MRI-defined white matter changes. It is also notable that this review has clarified the functional consequences of e2/e4 genotype. Across various neuropathology, imaging, cognition, and AD prevalence studies, e2’s potential protective effect was reduced greatly when combined with e4 and that e2/e4 was essentially as risk-promoting as e4. In this sense, it appears that the presence of the risky e4 allele “overrides” any protective effect that e2 might have on AD pathology. These findings posit further questions regarding the mechanism of e2 allele protection while also pointing to the limitations of e2 neuroprotection theory.

Many of the findings from our review were unexpected. We had hypothesized, albeit implicitly, that e2 would have wide-ranging protective effects, especially in cognition and *in vivo* MRI brain measures in the context of aging. Findings from various neuroimaging studies were mixed, and while there are some indications of preserved cortical thickness or hippocampal volume in e2 carriers, there is a lack of consensus on these topics, thus warranting further investigations. Beyond these reports there are a plethora of functional neuroimaging studies with disparate outcomes. Examination suggests that it would be difficult to state *a priori* whether a given outcome is advantageous or disadvantageous and thus those conclusions drawn appear to be *post-hoc*. Given e2’s strong association with AD risk, one might expect that e2 would also strongly associate with cognition, which is a key clinical marker for AD. However, we were surprised to find that there is a lack of consistency on e2’s association with cognition. Positive results, albeit marginal, were observed for older adults followed longitudinally for memory and general cognition. Findings differed by age- and clinical groups, indicating that e2’s mechanism toward preserved cognition may depend on various age-related brain changes. Current findings on e2’s neuroprotective role may have been also confounded by undetected AD pathology in many samples, even in the studies that were focused on “cognitively normal” older adults. In other words, are findings driven by subtle decline in the e3 group due to prodromal AD? Consistent with the idea that e2 is not a general, across the life span, cognitive enhancing variant, Conejero-Goldberg et al. (2014) found that expression of long-term potentiation (LTP) related genes was reduced in the post-mortem cortex of middle aged e2 carriers Trommer et al. (2004) and Conejero-Goldberg et al. (2011) found that LTP itself was reduced in APOE e2 TG mice. As LTP is a cellular marker of learning, this in and of itself may suggest limitations of cognitive enhancement or advantage. Small and inconsistent findings appeared to go beyond that expected due to some degree of mixed methodologies and small samples that represent e2 carriers. For example, it seemed plausible that the protective effect of e2 may have been confounded or inflated by methods that compared e2 with combined groups of e4 and e3 carriers. Results from these studies can be misleading given that APOE e4 is a well-established risk genotype. More accurate comparison can be made when e2 allele is compared with e3 homozygotes, the “neutral” genotype and thus demonstrate a valid protective effect. Nonetheless, in the more rigorous contrast, most studies that examined e2 only with e3 homozygotes found comparable or slightly more advantageous cognitive/brain outcomes in e2 carriers (exclusive of WMH).

## Conclusion

In conclusion, our comprehensive review of existing literature found circumscribed findings regarding the protective effect of APOE e2 on AD neuropathology (Table 1). However, current findings on e2’s protective role beyond AD neuropathology are mixed and the potential advantage of e2 allele is weakened by methodological heterogeneity, further studies are warranted to clarify the association between e2 and brain function in fMRI and cognition, along with underlying mechanisms. There is still a large gap in the literature regarding how e2’s reduced risk of AD neuropathologic markers does not transfer to reduced risk of brain morphometric and cognitive outcomes related to AD. While some studies suggest that various demographic (e.g., age and sex) and clinical (e.g., diabetes, hyperlipidemia, and MCI/AD) factors may play important roles in modifying e2’s effects, further large-scale studies with sufficient e2 sample and long follow-up durations may elucidate the function of e2. Furthermore, understanding the molecular mechanisms by which e2 confers protection and at what developmental stage will both advance and usefully constrain clinical research.

**TABLE 1:** Summary of findings.

Outcomes	Summary of findings	Comment
Longevity	Strong evidence for longevity in e2	E2 appears to have a strong effect, perhaps independent of its effects on AD.
Neuropathology	Circumscribed protective effects of e2 on AD-related neuropathologies (less accumulation of amyloid and tau aggregates)	E2 exhibits a protective effect with reduced Aβ, neuritic plaque, and NFT; however, e2 may promote risk for certain FTLDs and tauopathies, as well as cerebral amyloid angiopathy.
Cognition	Weak and inconsistent evidence for e2 effect	Stronger findings in longitudinal datasets and in clinical populations (MCI and AD), but findings are considered inconclusive due to heterogeneous methods.
**Neuroimaging**		
Structural MRI	Weak and inconsistent evidence for e2 effect	Stronger findings in longitudinal datasets and in clinical populations (MCI and AD), but findings are inconclusive due to heterogeneous methods.
Amyloid PET	Strong evidence for reduced PET Aβ with e2	Findings were similar across cross-sectional and longitudinal analyses, as well as in healthy controls and in MCI and AD.
White matter hyperintensities (WMH)	Strong evidence for increased WMH in e2	Findings were consistent with t other cerebrovascular tissue and vessel pathologies.
Diffusor tensor imaging (DTI)	Weak and inconsistent evidence for e2 effect	Very few studies have been conducted, making meaningful interpretations difficult.
Functional imaging	Weak and inconsistent evidence for e2 effect	Age and AD pathology may alter the relationship between e2 and functional connectivity.
CSF biomarkers	Strong evidence for the effect of e2 on lower CSF Aβ but inconsistent findings for CSF tau	Findings corroborated neuropathology literatures for Aβ, but not for tau. The reasons for this are obscure.

## Questions

1. How many years does e2 add to the lifespan? Is this increase associated with e2’s reduction in AD or is it partially independent? If it is the latter what are the mechanisms (e.g., is it an anti-frailty gene)?
2. What is the course of e2 associated cognition across the lifespan? Is it possible that it is disadvantageous early? Are the right cognitive instruments being used to capture any potential e2 effects?
3. What are molecular mechanisms of neuroprotection?
4. It is established that e2 reduces AD cortical amyloid and tau cortical histopathologies. Why then does it not reduce risk for CAA (and why does it increase risk for intra cerebral hemorrhage)? Insofar as it effects tau through a direct path, why also does it not have a protective effect on various tauopathies?
5. Little work has been done on e2 effects in ethnic groups other than Caucasians. This has been especially true for neuropathology. Sex effects have also been understudied.
6. Can gene editing techniques be applied to non-e2 SNPs in APOE exon 4 and so convert them to e2 alleles in embryos *in vitro*, and should they be from an ethical standpoint? Similarly, can AAV delivered APOE e2 to CSF have a favorable effect on AD related outcomes and at what age and to which genotype should such delivery be conducted?

## Author Contributions

HK contributed to the literature search/review and manuscript preparation and revision. DD contributed to the result interpretation and manuscript revisions. SC contributed to the manuscript preparation and revisions. TG contributed to the overall design of the manuscript, including literature searches and reviews and manuscript preparation and revision. All authors contributed to the article and approved the submitted version.

## Conflict of Interest

The authors declare that the research was conducted in the absence of any commercial or financial relationships that could be construed as a potential conflict of interest.

## Publisher’s Note

All claims expressed in this article are solely those of the authors and do not necessarily represent those of their affiliated organizations, or those of the publisher, the editors and the reviewers. Any product that may be evaluated in this article, or claim that may be made by its manufacturer, is not guaranteed or endorsed by the publisher.
