# Supplementary material for: Apolipoprotein E Genotype e2: Neuroprotection and Its Limits
Source: Front Aging Neurosci. 2022 Jul 14;14:919712. doi: 10.3389/fnagi.2022.919712 (PMC9329577; doi:10.3389/fnagi.2022.919712)
Supplement: Supplementary file 1 [file Table_1.pdf]

Supplemental Table 1. Summary of findings on APOE e2 and Cognition

**Cross-Sectional Findings**													
**Study**	**Population**	**Age**	**N/ ε2 n**	**Predictor**	**Comparisons**	***Global Cognition***	***Processing Speed***	***Working Memory***	***Executive Functions***	***Visual-Spatial***	***Verbal Fluency***	***Language***	***Memory***
Sinclair et al. (2017)(61)	UK	23-67	114/33	ε2/ε2, ε2/ε3	ε3/ε3		**NS**	**O**	**O**			**NS**	**O**
Marioni et al (2016)(64)	UK (GS:SFHS)	35-65	18337/ 2716 (111 ε2/ε2, 2194 ε2/ε3; 411 ε2/ ε4)	ε2/ε2, ε2/ε3, ε2/ ε4	Within ε2 groups, ε3/ε3, and ε4 (ε3/ε4, ε4/ε4), separately		**NS**				**NS**		**NS**

Lancaster et al. (2017)(63)	UK	45-55	66/16	ε2/ε2, ε2/ε3	ε3/ε3		**X**						
Greenwood et al. (2000)(62)	USA	50+, "middle -aged"	97/11	ε2/ε2, ε2/ε3	ε3/ε3 and ε4 group (ε2/ε4, ε3/ε4, ε4/ε4), separately		**NS**						
Allred et al. (2016)(65)	USA (DHS MIND)	Mean 65.9	1217/154	ε2/ε2, ε2/ε3	ε3/ε3	**X**	**NS**		**NS**			**X**	**X**
Alfred et al. (2014)(66)	UK (HALCyon, NCDS)	44-90+	23916/3085	ε2/ε2, ε2/ε3	ε3/ε3		**NS**				**NS**		**NS**
Shin et al. (2014)(139)	S. Korea (The	45-74	10371	ε2/ε2, ε2/ε3	ε3/ε3	**NS**							

	Namwon Study)												
Helkala et al. (1995)(140)	Finnish	Mean 73	916/76	ε2/ε2, ε2/ε3	ε2/ε4, ε3/ε4, ε4/ε4							**O**	**O**
Deary et al. (2004)(70)	UK (Lothian Birth Cohort)	Mean 79	462/66	ε2/ε3	ε3/ε3								**O**
Groot (2018)*(90)	Netherlands	Mean 69.2	180/36	ε2/ε3	ε3/ε3 and ε4 (ε3/ε4, ε4/ε4), separately		**X**		**X**			**X**	**O**
Gong (2020)(80)	USA (ADNI)	Mean 72.05	177/32	ε2/ε2, ε2/ε3	ε3/ε3 and ε4 (ε3/ε4, ε4/ε4), separately	**O**			**O**				**O**

Conejero-Goldberg (2014)(21)	USA (ADNI)		Mean age 75	31/5	ε2/ε2, ε2/ε3	ε3/ε3 and ε4 (ε3/ε4, ε4/ε4), separately				**NS**				**NS**
Kantarci (2012)(82)	USA (Mayo Clinic Study of Aging)		79 (76-83)	51/408	ε2/ε2, ε2/ε3	ε3/ε3 and ε4 (ε3/ε4, ε4/ε4), separately	**X**							
Bennett (2009)(67)	USA (ROS and MAP)		mean 89.3 for AD; 85.1 for No AD	74/536	ε2/ε2, ε2/ε3	ε3/ε3	**NS**	**NS**	**NS**	**NS**	**NS**	**NS**	**NS**	**NS**
**Longitudinal Findings**														
**Study**	**Population**	**F/U Duration**	**Age at (Baseline in y)**	**N/ ε2 n**	**Predictor**	**Comparisons**	***Global Cognition***	***Processing Speed***	***Working Memory***	***Executive Functions***	***Visual-Spatial***	***Semantic Fluency***	***Language***	***Memory***

Bunce et al. (2014)(141)	Astralia (PATH)	8 y	20-64	2013/35	ε2/ε2, ε2/ε3	ε3/ε4, ε4/ε4		**NS**	**NS**	**NS**				**NS**
Chiang et al. (2010)(93)	USA (ADNI)	2 y	55-90	134/27	ε2/ε2, ε2/ε3	ε3/ε3	**NS**							**NS**
Reas et al. (2019)(142)	USA (Rancho Bernardo Study)	Mean 7 y	44-99	1393/195	ε2/ε3	ε3/ε3 and ε4 (ε3/ε4, ε4/ε4), separately	**NS**			**O**				**NS**
Greenwood et al. (2000)(62)	USA (BIOCARD)	3- and 12- mo	33-85	139/not reported	ε2/ε2, ε2/ε3	ε3/ε3 and ε4 (ε3/ε4, ε4/ε4), separately		**O**						

Blair et al. (2005)(77)	USA (ARIC)	6 y	47-68	7895/ 1401	ε2/ε2, ε2/ε3	ε3/ε3		**O**				**NS**		**O**
Rajan et al. (2019)(73)	USA	8.9 y	65+	5807/ 48 ε2/ε2, 782 ε2/ε3, 203 ε2/ε4	ε2/ε2 vs. ε2/ε3 vs. ε2/ε4	ε3/ε3	**O**	**NS**						**O**
Staehelin et al. (1999)(72)	Switzerland (IDA)	2 y	65+	232/ 62	ε2/ε2, ε2/ε3, ε2/ε4	ε3/ε3 and ε4 (ε3/ε4, ε4/ε4), separately	**O**	**O**						**O**
Yaffe et al. (1997)(143)	USA (SOP)	6 y	65+	1750/259	ε2/ε2, ε2/ε3	ε3/ε3, ε3/ε4, ε4/ε4	**NS**	**O**	**NS**					

Henderson et al. (1995)(144)	Australia	3-4 y	70+	638/ (2 e22, 67 e23, 10 e24)	ε2/ε3+ ε2/ε4, ε2/ε2 separately	Within ε2 groups & ε3/ε3, ε3/ε4, ε4/ε4		**NS**					**NS**	**NS**
Blacker et al. (2007)*(78)	USA	5 y	71.38	107/16 in HC	ε2/ε2, ε2/ε4	ε3/ε3, ε3/ε4, ε4/ε4	**O**							
			72.90	235/36 in MCI	ε2/ε2, ε2/ε4	ε3/ε3, ε3/ε4, ε4/ε4	**NS**							
Helkala et al. (1996)(145)	Finnish	3 y	Mean 72	632/62	ε2/ε2, ε2/ε3	ε3/ε3, ε3/ε4, ε4/ε4	**NS**	**NS**		**NS**		**NS**		**O**
Shinohara (2016)(48)	USA (NACC)	3y	73	1,971/ 2,1531	ε2/ε2, ε2/ε3	ε3/ε3	**O**							
Grothe (2017)(97)	USA (ADNI)	2 y	mean age 73.0 in e2	572 HC and MCI 50	ε2/ε3	ε3/ε3								**NS**

Kim et al. (2017)(100)	S. Korea/subcortical vascular cognitive impairment	3 y	73+	72/11	ε2/ε2, ε2/ε3	ε3/ε3	**O**							
Wilson et al. (2002)(68)	USA (Religious Orders Study)	≤8 y	Mean age 74	669/86	ε2/ε2, ε2/ε3	ε3/ε3		**NS**	**NS**		**NS**			**O**
Bonner-Jackson et al. (2012)(146)	USA (ADNI)	2 y	74+	795/53	ε2/ε2, ε2/ε3	ε3/ε3, ε3/ε4, ε4/ε4	**O**	**NS**		**O**			**NS**	**O**
Martins et al. (2005)(79)	UK (OPTIMA Study); AD patients	6-month	Mean age mid-70's	218/24	ε2/ε2, ε2/ε3, ε2/ε4	ε3/ε3 and ε4 (ε3/ε4, ε4/ε4), separately	**O**							

Hyman et al. (1995)(36)	USA (Iowa 65+ Rural Health Study of the Elderly)	4-7 y	Mean 79	1899/296	ε2/ε2, ε2/ε3, ε2/ε4	ε3/ε3, ε3/ε4, ε4/ε4								**O**
Oveisgharan et al. (2018)	USA (ROP and MAP)	7-8 y	Mean 78.3	2151/ 301 (46 ε2/ε4)	ε2/ε4, ε2 groups (ε2/ε2, ε2/ε3)	ε3/ε3, ε2 groups (ε2/ε2, ε2/ε3), ε4 groups ε3/ε4, ε4/ε4), separately	**O**							
Schiepers et al. (2012)(71)	UK (Lothian Birth Cohort)	8 y	79-87	501/78	ε2/ε2, ε2/ε3, ε2/ε4	ε3/ε3, ε3/ε4, ε4/ε4				**NS**				**NS**

Lindahl-Jacobson (2013)(69)	Denmark (Danish 1905 Birth Cohort)	2y, 5y, 7y	92-93	1651/163	ε2/ε2, ε2/ε3	ε3/ε3	**O**							

O=protective role of APOE e2; **Δ**= marginally significant; **NS**= Non-significant association between APOE e2 and cognition; **X**= adverse role of APOE e2

Supplemental Table 2. Summary of Findings on APOE e2 and Imaging Biomarkers

**Cross-Sectional Findings**												
**Study**	**Population**	**Participant Age (y)**	**N/ ε2 n**	**Predictor**	**Comparison**	***Cortical Thickness***	***Ventricles***	***Regional***	***Hippocampal Vol.***	***Amyloid B plaques***	***WM integrity***	***WMH***
Alexopoulos (2011)(147)	Germany	24.7	33/17	ε2/ε2, ε2/ε3	ε3/ε3, ε3/ε4				**O**			
Fennema-Notestine (2011)(86)	USA (VETSA)	ages 51-59	482/ 67	ε2/ε3	ε3/ε3			**O**	**Δ**			
Konishi (2016)(148)	Canada	18-37	37/5	ε2/ε2, ε2/ε3	ε3/ε3 and ε4 (ε3/ε4 and ε4/ε4)				**O**			

Raz (2012)(109)	USA	44-77 (mean 58.89)	144/19	ε2/ε2, ε2/ε3	ε3/ε3 and ε3/ε4, ε4/ε4							**X**
Bunce (2012)(92)	Australia (PATH)	Middle-aged (44-48); Older adults (64-68)	627/86	ε2/ε2, ε2/ε3	ε3/ε3 and ε4 (ε3/ε4, ε4/ε4)			**NS**				
Morris (2010)(95)	USA		45-88	241/ 3 e2/e2; 26 e2/e3, 5e2/e4	ε2/ε2, ε2/ε3, ε2/ε4	ε3/ ε3, ε3/ ε4, ε4/ ε4					**O**	
Westlye (2012)(113)	Norway (Norwegian Cognitive Neuro Genetics)	Mean 47.6 (21-69 y)	203/30	ε2/ε3	ε3/ε3 and ε3/ε4						**X**	

Schmidt (1997)(110)	Austria (Austria Stroke Prevention Study)	50-75	280/34	ε2/ε3	ε3/ε3							**X**
Salvado (2019)(106)	Spain; Alzheimer and Families (ALFA) Study	51-64 (mean 58)	109/561	ε2/ε3	ε3/ε3							**O**
Liu (2010)*(83)	Europe	65+	*105/12 in HC*	ε2/ε2, ε2/ε3	ε3/ε3 and ε4 (ε3/ε4, ε4/ε4)	**NS**	**NS**	**NS**	**NS**			
			109/10 in MCI	ε2/ε2, ε2/ε3	ε3/ε3 and ε4 (ε3/ε4, ε4/ε4)	**O**	**O**	**O**	**Δ**			

			115/4 in AD	ε2/ε2, ε2/ε3	ε3/ε3 and ε4 (ε3/ε4, ε4/ε4)	**O**	**O**	**O**	**Δ**			
Serra-Grabulosa (2003)*(84)	Spain	mean 65.5	50/14	ε2/ε3	ε3/ε3 and ε4 (ε2/ε4, ε3/ε4, ε4/ε4)	**NS**	**NS**		**NS**			
Chiang (2012)(114)	USA	68 (49-90)	37/19	ε2/ε3	ε3/ε3						**O**	
Groot (2018)(90)	Netherlands	Mean 69.2	180/36	ε2/ε3	ε3/ε3 and ε4 (ε3/ε4, ε4/ε4)	**Δ**						**X**
Chen (2015)(124)	China	69.3 in e2 HC	100/16 in HC	ε2/ε3	ε3/ε3 and ε4 (ε3/ε4, ε4/ε4)			**Δ**				

		64.8 in e2 aMCI	85/9 in aMCI	ε2/ε3	ε3/ε3 and ε4 (ε3/ε4, ε4/ε4)			**O**				
Den Heijer (2002)(88)	Netherlands (Rotterdam Scan Study)	aged 60-90; mean age 72, 7	949/120	ε2/ε2, ε2/ε3	ε3/ε3			**NS**	**NS**			
Fan (2010)(89)	USA (ADNI)	mean 74.45, 5.97 in e2 group	164/21	ε2/ε2, ε2/ε3	ε3/ε3 and ε4 (ε3/ε4, ε4/ε4)			**O**				
Hostage (2013)(87)	USA (ADNI)	Mean 75.2 in HC	198/26 in HC	0 ε2 (ε3/ε3, ε3/ε4, ε4/ε4) vs. 1 ε2 (ε2/ε3, ε2/ε4) vs. 2 ε2 (ε2/ε2)					**Δ**			
		Mean 76.9 in MCI	321/14 in MCI	0 ε2 (ε3/ε3, ε3/ε4, ε4/ε4) vs. 1 ε2 (ε2/ε3, ε2/ε4) vs. 2 ε2 (ε2/ε2)					**NS**			

		Mean 75.5 in AD	143/4 in AD	0 ε2 (ε3/ε3, ε3/ε4, ε4/ε4) vs. 1 ε2 (ε2/ε3, ε2/ε4) vs. 2 ε2 (ε2/ε2)					**NS**			
Khan (2017)(85)	Multi cohort (AD and normal aging dataset)	Mean 75.8 in HC	375/17	ε2/ε3, ε2/ε3	ε3/ε3 and ε4 (ε3/ε4, ε4/ε4)				**NS**			
		Mean 75.6 in MCI	522/29	ε2/ε3, ε2/ε3	ε3/ε3 and ε4 (ε3/ε4, ε4/ε4)				**NS**			
		Mean 78.1 in AD	512/66	ε2/ε3, ε2/ε3	ε3/ε3 and ε4 (ε3/ε4, ε4/ε4)				**NS**			
Lim (2017)(96)	USA (ADNI)	mean age 75.9 for AB+ e2	595/59	ε2/ε2, ε2/ε3	ε3/ε3 and ε4 (ε2/ε4, ε3/ε4, ε4/ε4)					**O**		

Conejero-Goldberg (2014)(21)	USA (ADNI)	Mean age 75	31/5	ε2/ε2, ε2/ε3	ε3/ε3 and ε4 (ε3/ε4, ε4/ε4)			**Δ**				
Grothe (2017)(97)	USA (ADNI)	mean age 73.0 in e2	572 HC and MCI/50	ε2/ε3	ε3/ε3					**O**		
Gong (2020)(80)	USA (ADNI)	Mean 72.05	177/32	ε2/ε2, ε2/ε3	ε3/ε3 and ε4 (ε3/ε4, ε4/ε4)			**NS**	**O**			
Luo (2017)(108)	USA (ADNI)	74	178/34	ε2/ε2, ε2/ε3	ε3/ε3 and ε3/ε4							**X**
Kantarci (2012)(82)	USA (Mayo Clinic Study of Aging)	79 (76-83)	51/408	ε2/ε2, ε2/ε3	ε3/ε3 and ε4 (ε3/ε4, ε4/ε4)					**O**		

Longitudinal Findings													
**Study**	**Population**	**F/U Duration**	**ParticipantAge at Baseline (y)**	**N/ ε2n**	**Predictor**	**Comparisons**	***Cortical***	***Ventricles***	***Regional***	***Hippocampal***	***Amyloid B***	***FA and MD***	***WMH***
Roe (2018)(91)	USA	6.2 y	42-90 (mean 67.6)	664/82	ε2/ε2, ε2/ε3, ε2/ε4	ε3/ε3, ε3/ε4, ε4/ε4					**O**		
Chiang (2010)(93)	USA (ADNI)	2y	55-90; mean 79 SD 4.6	134/27	ε2/ε2, ε2/ε3	ε3/ε3	**O**			**O**			
Kim et al. (2017)(100)	S. Korea *	3 y	73+		ε2/ε2, ε2/ε3	ε3/ε3	**O**				**O**		
Hall (2019)(99)	Finland (Vanta	5.6 y	85+; Healthy at baseline	163/26	ε2/ε2, ε2/ε3, ε2/ε4	ε3/ε3, ε3/ε4, ε4/ε4					**O**		**X**

	a 85+ Study)												

O=statistically significant protective role of APOE e2; **Δ**= trend level significance for e2 neuroprotection; **NS**= Non-significant association between APOE e2 and imaging markers; **X**= adverse role of APOE e2

*Study participants included clinical samples (MCI and/or AD)
